# Solvent-dependent complex reaction pathways of bromoform revealed by time-resolved X-ray solution scattering and X-ray transient absorption spectroscopy

**DOI:** 10.1063/1.5132968

**Published:** 2019-12-24

**Authors:** Qingyu Kong, Dmitry Khakhulin, Ilya A. Shkrob, Jae Hyuk Lee, Xiaoyi Zhang, Jeongho Kim, Kyung Hwan Kim, Junbeom Jo, Jungmin Kim, Jaedong Kang, Van-Thai Pham, Guy Jennings, Charles Kurtz, Rick Spence, Lin X. Chen, Michael Wulff, Hyotcherl Ihee

**Affiliations:** 1 Synchrotron Soleil, L'Orme des Merisiers, St. Aubin, 91192 Gif-sur-Yvette, France; 2 School of Physics Science and Information Technology, Liaocheng University, Shandong Key Laboratory of Optical Communication Science and Technology, Liaocheng University, Liaocheng 252059, China; 3 European XFEL GmbH, Holzkoppel 4, D-22869 Schenefeld, Germany; 4 Chemical Sciences and Engineering Division, Argonne National Laboratory, 9700 S. Cass Ave., Argonne, Illinois 60349, USA; 5 Pohang Accelerator Laboratory, Pohang 37673, South Korea; 6 X-ray Science Division, Argonne National Laboratory, 9700 S. Cass Ave., Argonne, Illinois 60349, USA; 7 Department of Chemistry, Inha University, Incheon 22212, South Korea; 8 Department of Chemistry, Pohang University of Science and Technology (POSTECH), Pohang 37673, South Korea; 9 Department of Chemistry and KI for the BioCentury, Korea Advanced Institute of Science and Technology (KAIST), Daejeon 34141, South Korea; 10 Center for Nanomaterials and Chemical Reactions, Institute for Basic Science (IBS), Daejeon 34141, South Korea; 11 Department of Chemistry, Northwestern University, 2145 Sheridan Rd., Evanston, Illinois 60208, USA; 12 European Synchrotron Radiation Facility, BP 220, F-38043 Grenoble Cedex, France

## Abstract

The photochemical reaction pathways of CHBr_3_ in solution were unveiled using two complementary X-ray techniques, time-resolved X-ray solution scattering (TRXSS) and X-ray transient absorption spectroscopy, in a wide temporal range from 100 ps to tens of microseconds. By performing comparative measurements in protic (methanol) and aprotic (methylcyclohexane) solvents, we found that the reaction pathways depend significantly on the solvent properties. In methanol, the major photoproducts are CH_3_OCHBr_2_ and HBr generated by rapid solvolysis of *iso-*CHBr_2_-Br, an isomer of CHBr_3_. In contrast, in methylcyclohexane, *iso*-CHBr_2_-Br returns to CHBr_3_ without solvolysis. In both solvents, the formation of CHBr_2_ and Br is a competing reaction channel. From the structural analysis of TRXSS data, we determined the structures of key intermediate species, CH_3_OCHBr_2_ and *iso*-CHBr_2_-Br in methanol and methylcyclohexane, respectively, which are consistent with the structures from density functional theory calculations.

## INTRODUCTION

An understanding of reaction mechanisms at atomic and electronic levels is one of the central themes in chemistry; many efforts have been devoted toward that goal with advances in chemical reaction dynamics. In particular, the development of time-resolved techniques has contributed to revealing mechanisms for various photochemical reactions in the gas phase and condensed media. Nevertheless, even for reactions of small molecules with simple chemical structures, it is still challenging to identify all of the major reaction intermediates along reaction pathways. One such example is the photochemistry of bromoform, CHBr_3_. Upon ultraviolet (UV) excitation, CHBr_3_ dissociates to generate reactive bromine, which effectively depletes ozone in the troposphere and/or the stratosphere.^1,2^ Due to both fundamental interest and environmental importance, photochemistry of CHBr_3_ has been studied intensively using time-resolved optical spectroscopy.^3–11^ However, the comprehensive photochemical reaction mechanisms of CHBr_3_ have not been completely understood, mainly because this small molecule with an ostensibly simple structure exhibits unusually complex reaction pathways.

In the gas phase, upon excitation at UV wavelengths, the primary reaction channel of CHBr_3_ is the cleavage of the C–Br bond to produce a CHBr_2_ radical and a Br atom,

$${\text{CHBr}}_{3}+hv\to {\;\text{CHBr}}_{2}+\;\text{Br}.$$ (R1)

Subsequently, secondary dissociation of the CHBr_2_ radical occurs to generate CBr + HBr or CHBr + Br (see Table S1).^9,10^ Direct elimination of Br_2_ is another possible reaction channel (CHBr_3_ + *hv* → CHBr + Br_2_).^11^

In the condensed phase, studies were focused on the dynamics of the *iso*-CHBr_2_-Br, which can be formed through roaming-mediated isomerization in the excited state^6^ or solvation cage-induced geminate recombination of CHBr_2_ and Br,^3–6^

$${\text{CHBr}}_{3}+hv\to \mathrm{iso}-{\text{CHBr}}_{2}-\text{Br}.$$ (R2)

Other short-lived intermediates or stable photoproducts, such as Br and Br_2_ that react directly with O_3_ (Br_2_ + *hv* → 2Br, Br + O_3_ → BrO + O_2_), have been rarely reported in solution; it might have been difficult to globally detect all the photoproducts, especially irreversible and stable species, due to limited time ranges (up to only a few nanoseconds) in most of the previous time-resolved spectroscopic studies. In addition, the photoproducts can further react with the surrounding solvent molecules in various ways, as exemplified by the Wohl-Ziegler reaction, in which the hydrogen in a solvent molecule is abstracted by the bromine atom.^7^ An additional reaction can occur when the solvent is a nucleophile. For example, it was reported by Kwok *et al.*^3^ that *iso-*CHBr_2_-Br formed by photolysis of CHBr_3_ in aqueous solution undergoes hydrolysis, resulting in complex chemistry.

Although time-resolved optical spectroscopy is highly sensitive to the formation of reaction intermediates, even for solution samples at low concentrations, it provides only limited structural insight in the reaction intermediates. In contrast, time-resolved X-ray solution scattering (TRXSS), which is also known as time-resolved X-ray liquidography (TRXL), is highly sensitive to the details of the molecular structure. TRXL can provide direct information on changes in the global molecular structure, which cannot be directly extracted from time-resolved optical spectroscopy, because X-rays are scattered off all atomic pairs present in sample molecules and the scattering pattern contains direct information on the distances of all atomic pairs.^12–50^ Complementary to TRXL, X-ray transient absorption (XTA) spectroscopy can provide the dynamics of the electronic structure and local geometric structure around light-absorbing atoms by probing the excitation of core electrons to modified valence electron orbitals.^51–54^ These two complementary techniques can be combined together to obtain more complete information on the reaction dynamics as was demonstrated in recent studies.^13,55^

**FIG. 1.**
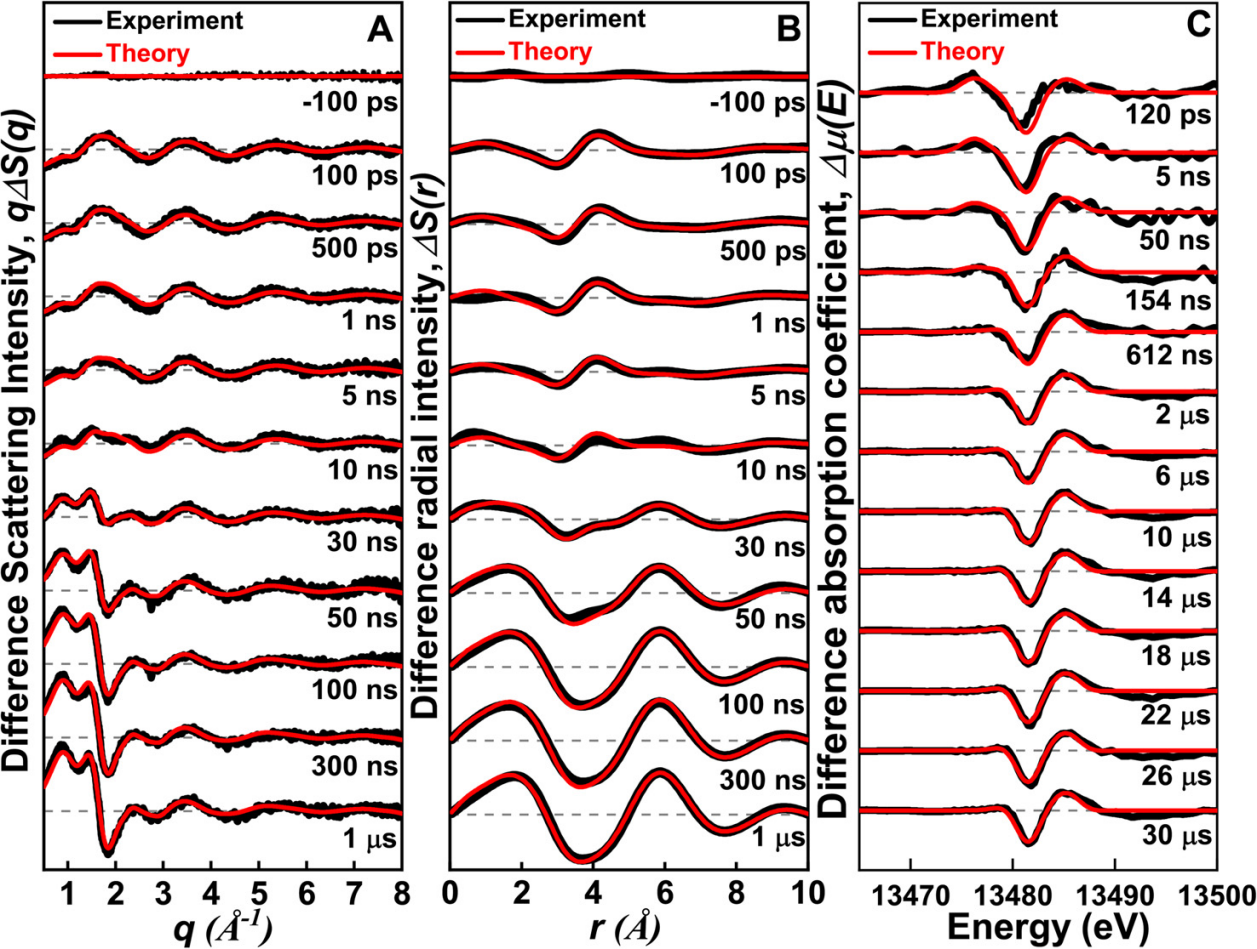
TRXL and XTA signals measured for CHBr_3_ in methanol after 267 nm laser excitation. (a) Difference scattering curves, *q*Δ*S*(*q*), measured at various delay times (black) and their theoretical fits (red) from a global fitting analysis described in the text. (b) Difference radial distribution functions, *r*Δ*S*(*r*), obtained by the sine-Fourier transform of *q*Δ*S*(*q*) shown in (a). (c) XTA signals measured at various delay times. Experimental XTA spectra (black) were fit by theoretical XTA spectra (red) calculated from putative reaction intermediates.

**FIG. 2.**
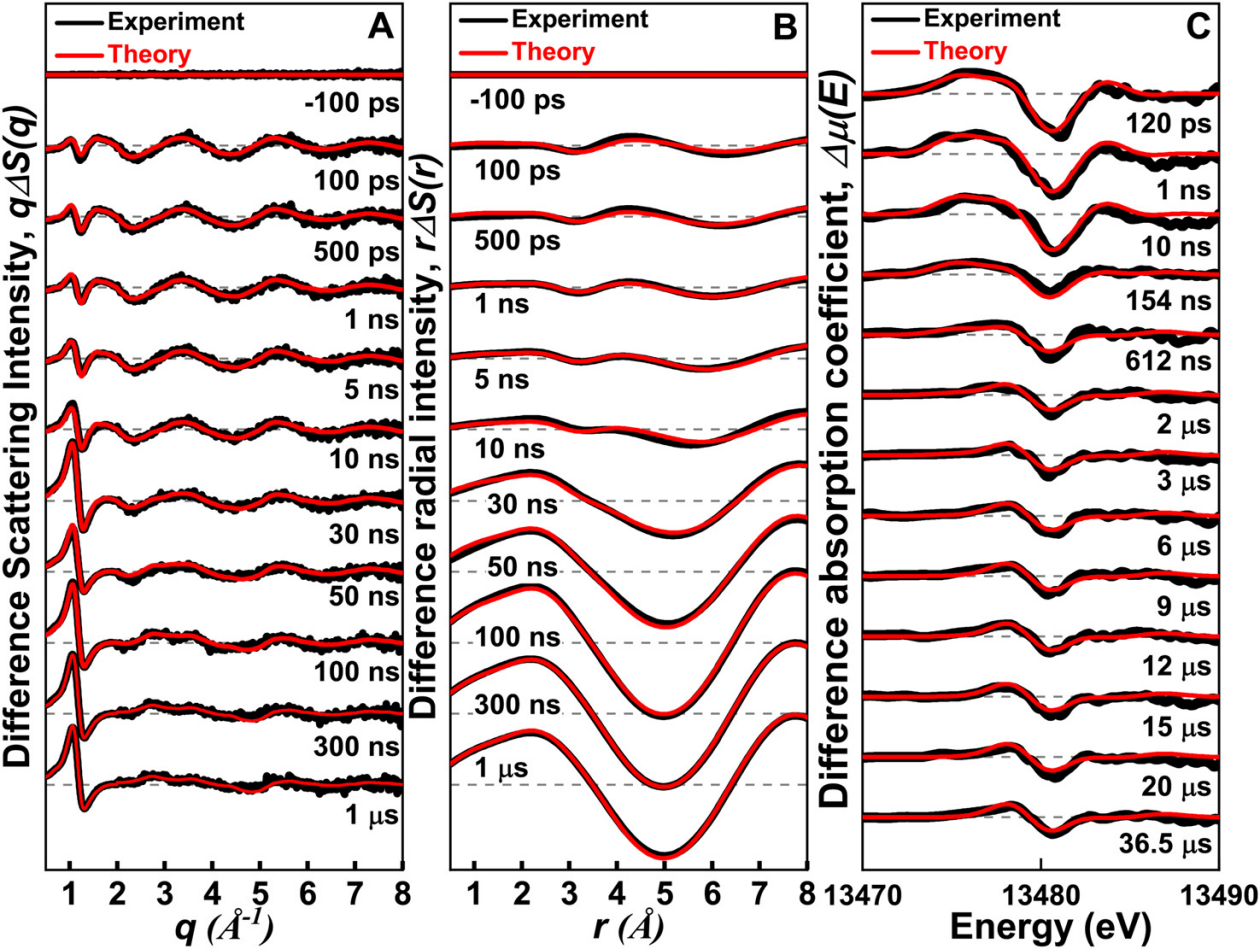
TRXL and XTA signals measured for CHBr_3_ in methylcyclohexane after 267 nm laser excitation. (a) Difference scattering curves, *q*Δ*S*(*q*), measured at various delay times (black) and their theoretical fits (red) from a global fitting analysis described in the text. (b) Difference radial distribution functions, *r*Δ*S*(*r*), obtained by the sine-Fourier transform of *q*Δ*S*(*q*) shown in (a). The low *q* signals from 100 ps to 30 ns, and from 50 ns to 1 *μ*s in (A), are divided by 3 and 6, respectively, for clarity. (c) XTA signals measured at various delay times. Experimental XTA spectra (black) were fit by theoretical XTA spectra (red) calculated from a global fitting analysis.

In this work, we applied TRXL and XTA to the photochemical reaction of CHBr_3_ in methanol and methylcyclohexane (see Figs. 1 and 2). By simultaneously analyzing the datasets from TRXL and XTA, we characterized the dynamics of geometric and electronic structures to completely describe the reaction dynamics of CHBr_3_ in solution. The results revealed the rich photochemistry of CHBr_3_ including the formation of Br and Br_2_ that directly relate to ozone depletion and solvent-dependent reaction pathways. Stable photoproducts, CH_2_Br_2_, BrCH_2_OH, C_2_H_2_Br_4_, *cis*-C_2_H_2_Br_2_, and *trans*-C_2_H_2_Br_2_ were also observed and characterized using gas chromatography mass spectrometry (GCMS) and NMR spectroscopies.

## RESULTS AND DISCUSSION

### TRXL measurement in methanol

To follow the photochemical reaction dynamics of CHBr_3_, we collected a series of TRXL data at various delay times as shown in Fig. 1(a). The experimental difference scattering curves, *q*Δ*S*(*q*), were obtained by subtracting the reference scattering signal measured at a negative time delay (–3 ns) from the scattering signals measured at positive delay times. It can be seen in Fig. 1(a) that the oscillatory features of the difference signal at positive delay times change considerably with time, reflecting the structural changes of the reacting molecules.

The total scattering signal S(*q*) from liquid can be described by the Fourier transform of the statistical correlation between scattering sites, g(*r*),^56^ which can be expressed as the sum of contributions from the solute, solvent, and solute-solvent interaction (that is, the solvation cage). To explain the measured difference scattering signal, we fitted the experimental data at various delay times using theoretical scattering curves, which are a linear sum of these three contributions, as shown in Fig. S1 in the supplementary material. For the solute species, all possible reaction pathways were considered as shown in Table S2 in the supplementary material. Specifically, we considered the following solute species: CHBr_3_, CHBr_2_, CHBr_2_^+^, Br, CBr_2_, CHBr, iso-CHBr_2_-Br, Br_2_, CH_3_OCHBr_2_, HBr, CH_2_Br_2_, (*anti- and gauche-*) C_2_H_2_Br_4_, (*trans- and cis-*) C_2_H_2_Br_2_, BrCH_2_OH, and CH_3_Br. The geometries of these species were optimized using density functional theory (DFT) calculations, as shown in Fig. S2. The data analysis is described in Secs. 2 and 3 in the supplementary material and Fig. S1, while its detailed description can be found in previous reports.^12,15,19^

Although the difference scattering curves, *q*Δ*S*(*q*,*t*), shown in Fig. 1(a) constitute a complete “fingerprint” of the structural rearrangements, the difference radial distribution functions (RDFs), *r*Δ*R*(*r*,*t*), in real space, shown in Fig. 1(b), provide a more intuitive picture of the changes in the molecular structure. The difference RDF represents the change in the atom-atom pair distribution function during the course of the reaction and thus is a measure of the change in radial electron density around an (average) excited atom as a function of interatomic distance *r*, weighted by the X-ray form factor. As shown in Fig. 1(b), several positive and negative peaks appear and evolve over time. Unequivocal peak assignments can be made by decomposing the signal into the contributions of the solute, solvent, and solvation cage as illustrated in Fig. S1. The negative peak at 3.25 Å appears at early delay times and can be attributed to the depletion of the Br···Br interatomic pairs in CHBr_3_. Subsequently, strong positive (at ∼5.5 Å) and negative (at ∼4 Å and ∼7 Å) peaks become dominant after 30 ns, and can be ascribed to the changes in the intermolecular distances of solvent molecules due to thermal expansion of the solution.^19^

We performed a structural analysis of the difference scattering curve at 100 ps as shown in Fig. S3, and the analysis result shows that reaction (R1) fits the data much better than reaction (R2), which was postulated to be a major reaction channel in the condensed phase.^3–6^ As can be seen in Fig. S3, the inclusion of *iso-*CHBr_2_-Br deteriorates the fitting quality significantly. When both reactions (R1) and (R2) were included in the analysis, the contribution of Reaction R2 converged to zero.

### XTA measurement in methanol

With 267 nm excitation, various reaction channels are energetically allowed as shown in Table S1, and thus all of them were considered in the analysis of TRXL data. Because light atoms (C and H) have much lower scattering intensities than heavy atoms (Br), TRXL is less effective for distinguishing chemical species that have the same heavy atoms but different light atoms. For example, as shown in Fig. S4, TRXL cannot unambiguously distinguish Reaction R1 from another reaction, CHBr_3_ → CBr_2_ + HBr. To complement such limitation of TRXL and obtain electronic structural dynamics, we performed XTA measurement on the photochemical reaction of CHBr_3_ because the method is sensitive to the change in electronic and local geometric structures around the X-ray absorbing atoms. Considering that the Br of CHBr_2_ and free Br atoms have unrestricted open electron shells while the Br of CBr_2_ and HBr are closed electron shell species, the two reaction channels should yield distinctly different XTA signals at the Br K-edge.

**FIG. 3.**
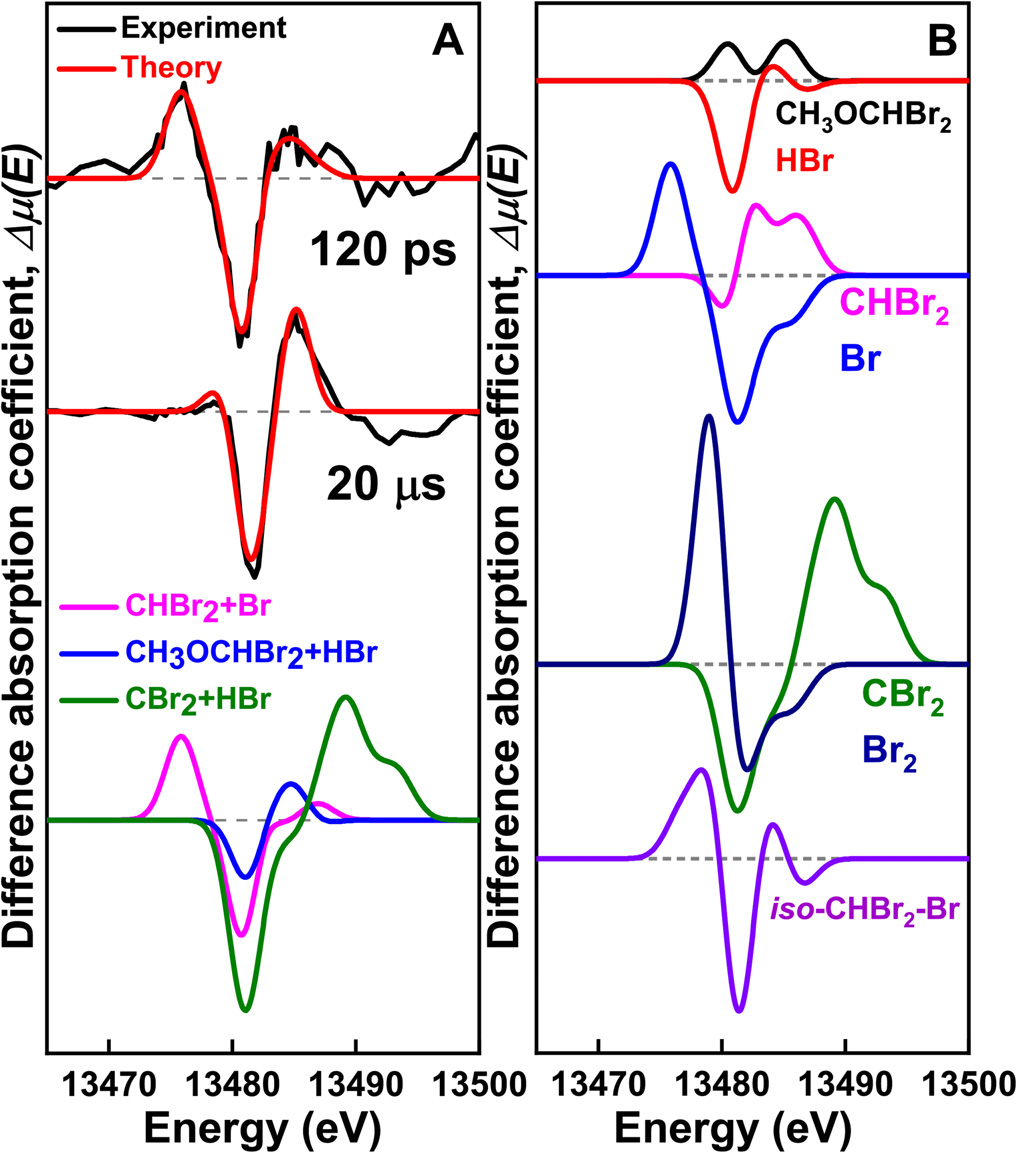
XTA signals measured for CHBr_3_ in methanol after 263 nm laser excitation. (a) (Top) XTA spectrum at 120 ps (black) fitted by the calculated spectra (red) considering two reaction channels: (R1) (CHBr_3_ → CHBr_2_ + Br) and (R3) (*iso*-CHBr_2_-Br + CH_3_OH → CH_3_OCHBr_2_ + HBr). (Middle) XTA spectrum at 20 *μ*s (black) compared with the calculated spectra (red) for (R3). (Bottom) Calculated spectra for (R1) (magenta), (R3) (blue) and CHBr_3_→ CBr_2_ + HBr (green). (b) Calculated XTA signals of chemical species considered for global fitting.

The Br K-edge XTA spectra in the X-ray absorption near edge structure (XANES) region for CHBr_3_ in methanol were measured at various delay times from 263 nm excitation, as shown in Figs. 1(c) and 3(a). A positive peak at 13 475 eV is observed at 120 ps, which corresponds to the absorption feature of Br (1s → 4p transition),^57^ providing a direct evidence for the formation of Br upon laser excitation of CHBr_3_. This feature disappears at later delay times, indicating the participation of Br in the formation of other species. It can be seen that the negative peak at 13 480 eV is shifted to higher energies over time while its intensity remains constant in the entire time range of our measurement (see Fig. S5). The negative peak arises from the depletion of ground-state molecules induced by photoexcitation. The blue shift of the negative peak with a nearly constant intensity suggests that, at late delay times, reaction intermediates formed at the onset of the reaction are transformed into other intermediate species rather than returning to the ground state.

To extract the structural dynamics of transient intermediates, the experimental XTA spectra were fitted with the theoretical XANES spectra calculated from putative reaction intermediates [see Fig. 3(b)]. In particular, we identified the intermediates by fitting the XTA signal at 120 ps with reaction (R1) and CHBr_3_ → CBr_2_ + HBr, which exhibit distinctly different XTA spectra as shown in Fig. 3(a). In fact, in the fitting analysis of the 120 ps spectrum, the contribution of the latter channel (CHBr_3_ → CBr_2_ + HBr) converges to zero, confirming that CHBr_3_ dissociates into the CHBr_2_ radical and Br, rather than CBr_2_ and HBr. The formation of CHBr_2_ was confirmed by applying electron paramagnetic resonance (EPR) spectroscopy to a photolyzed solution of CHBr_3_ in methanol frozen at 77 K (Sec. 5 in the supplementary material). A previous transient Raman spectroscopic study on the photochemical reaction of CHBr_3_ in methanol also showed that the major product at 5 ns delay time is the CHBr_2_ radical.^58^ This result clarifies the uncertainty regarding the identities of intermediates that cannot be unequivocally resolved by the analysis of TRXL data.

**FIG. 4.**
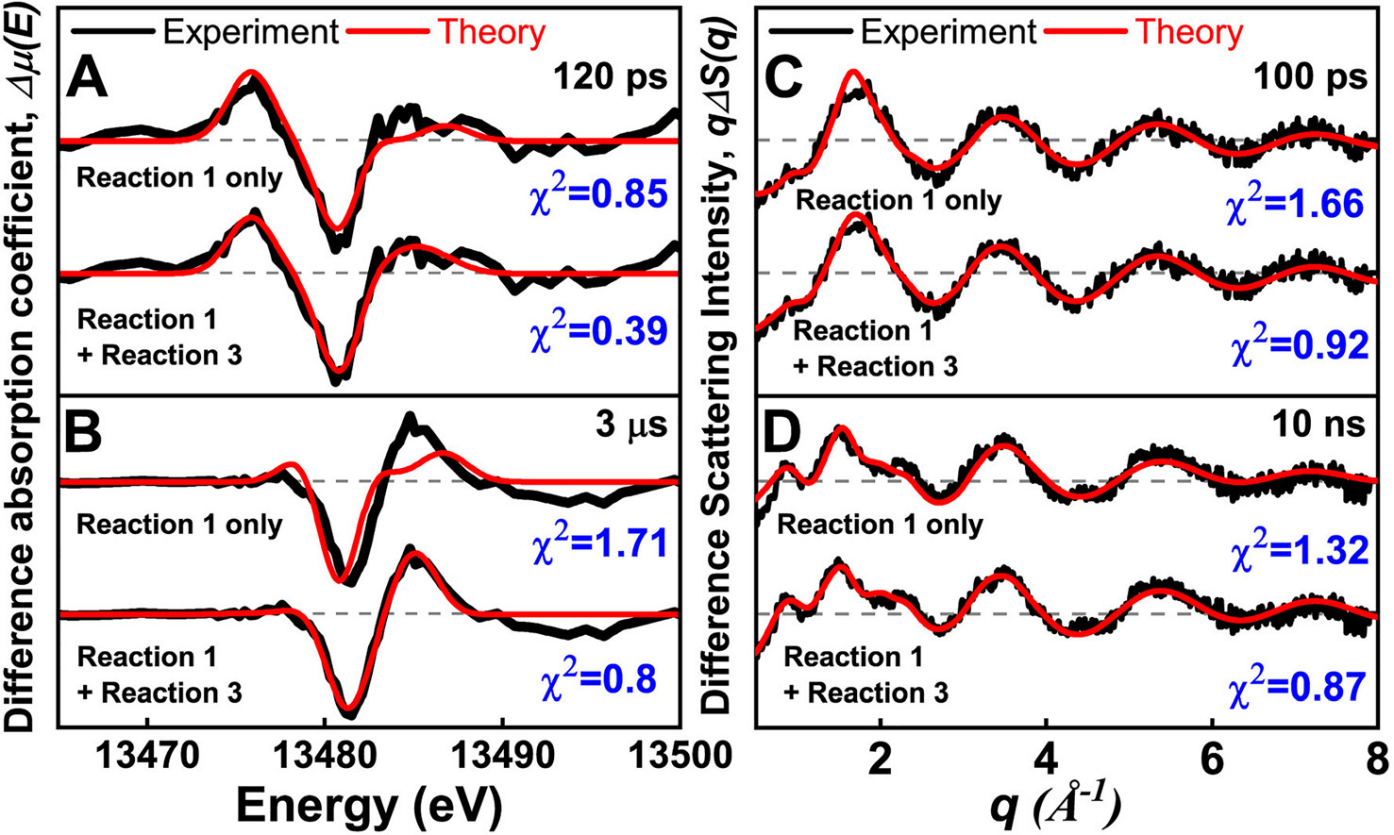
Fitting of the XTA and TRXL data of CHBr_3_ in methanol. (a) and (b) XTA spectra obtained at (a) 120 ps and (b) 3 *μ*s. (c) and (d) TRXL curves obtained at (c) 100 ps and (d) 10 ns. For both analyses, we compared two cases: (i) reaction (R1) alone and (ii) a combination of reactions (R1) and (R3). It can be seen that the combination of reactions (R1) and (R3) gives better quality fits to the experimental XTA and TRXL data.

Meanwhile, in accordance with the result of the TRXL analysis, XTA confirms that *iso*-CHBr_2_-Br is not observed at 120 ps, as shown in Fig. S6. While CHBr_2_ and Br are identified as the intermediates at the earliest delay time, the analysis of the XTA spectrum at 120 ps shows that reaction (R1) alone cannot produce a perfect fit to the experimental data as can be seen in the upper plot in Fig. 4(a), indicating that other intermediates are also produced at the early stage of the photochemical reaction of CHBr_3_. The discrepancy between the experimental and theoretical spectra becomes more pronounced at late delay times [for example, at 3 *μ*s, as can be seen in the upper plot in Fig. 4(b)]. By considering all the possible reaction pathways listed in Table S2, we found that the following solvolysis reaction is required to achieve good agreement between the experimental and simulated XTA spectra on time scales from picoseconds to microseconds, as can be seen in the lower plots in Figs. 4(a) and 4(b),

$$\mathrm{iso}-{\text{CHBr}}_{2}-\text{Br}\;+{\;\text{CH}}_{3}\text{OH}\;\to {\;\text{CH}}_{3}{\text{OCHBr}}_{2}+\;\text{HBr}.$$ (R3)

Inspired by the result of this XTA data analysis, we reanalyzed the TRXL data by considering both Reaction R3 and Reaction R1. As a result, the fitting qualities of the TRXL data improve significantly at both early and late delay times, as can be seen in Figs. 4(c) and 4(d). The solvolysis reaction of *iso*-CHBr_2_-Br [reaction (R3)] is highly exothermic (see Table S2), and a similar solvolysis reaction was observed in a transient resonance Raman spectroscopic study of CHBr_3_ in water.^3^ The reaction between CHBr_2_ and one solvent molecule (CHBr_2_ + CH_3_OH → CH_3_OCHBr_2_ + H) can also generate CH_3_OCHBr_2_, but it is an endothermic reaction with an energy barrier of 130.43 kJ/mol (Table S2) and thus is less favorable. In fact, the formation of HBr from the solvolysis reaction was confirmed by reacting the photolyzed solution of CHBr_3_ in methanol with silver nitrate, which yields an insoluble AgBr precipitate.

**FIG. 5.**
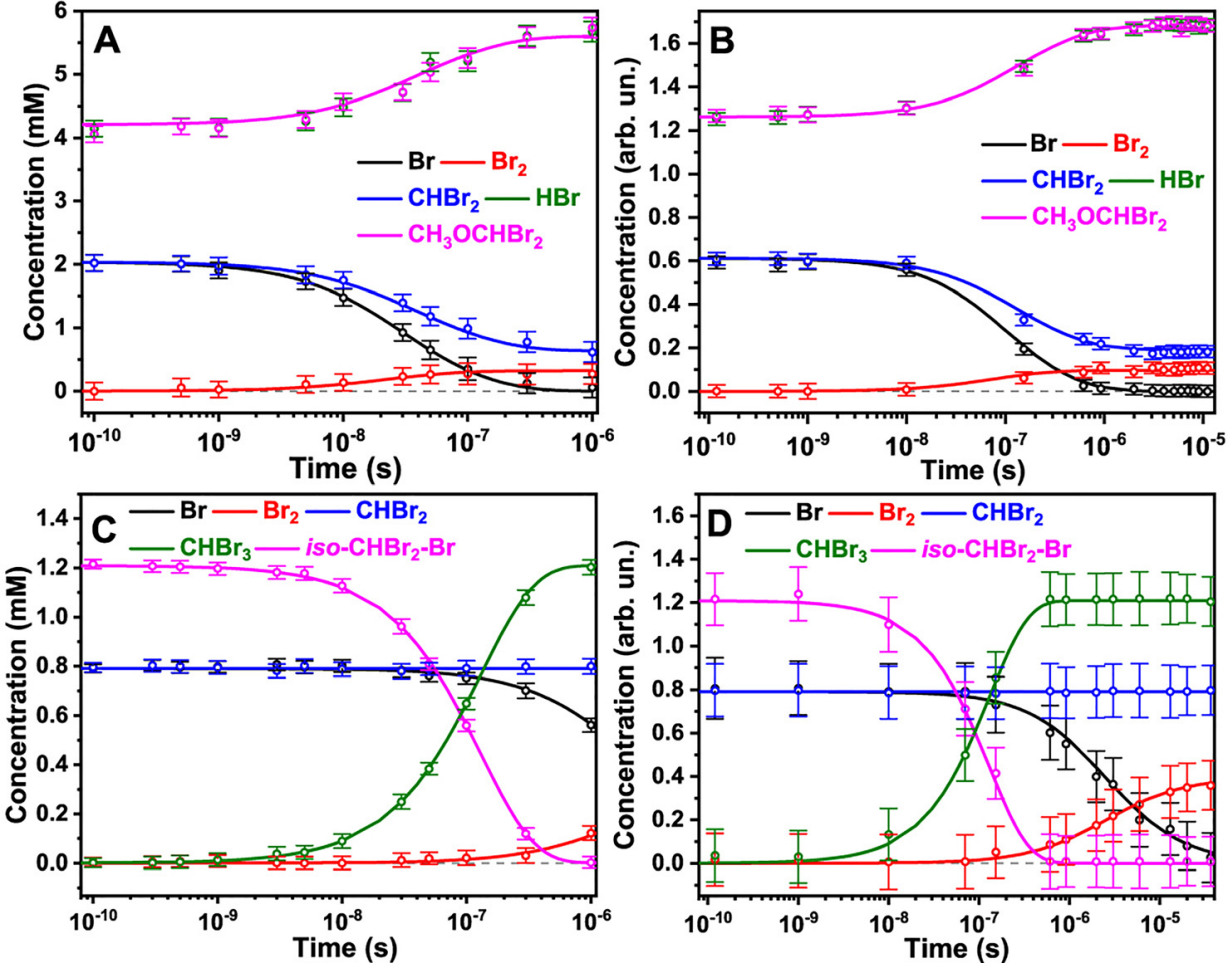
Kinetic of various intermediates in the photochemical reaction of CHBr_3_ in methanol and methylcyclohexane. (a) and (b) Time-dependent population changes of reaction intermediates and photoproducts in methanol determined by the simultaneous global fit analysis of (a) TRXL data and (b) XTA data. The curves corresponding to HBr and CH_3_OCHBr_2_ are overlapped. (c) and (d) Time-dependent population changes of reaction intermediates and photoproducts in methylcyclohexane determined by the simultaneous global fit analysis of (c) TRXL data and (d) XTA data. The error bar at each data point illustrates the standard error from 50 [(a)–(c)] and 30 (d) measurements, respectively. The solid lines were obtained from the global fit analysis.

To investigate the photochemical reaction pathways of CHBr_3_ in detail, we determined the time-dependent concentrations of major intermediates and products from a combined global fitting (GF) analysis of the TRXL and XTA data as shown in Figs. 5(a) and 5(b), respectively. The combined global fitting approach, which is described in detail in Sec. 3 in the supplementary material, is a fully consistent and complete data analysis framework because it takes advantage of both the local electronic element-specific sensitivity of XTA and the global structural and thermodynamic sensitivity of TRXL. According to the kinetics determined from the TRXL data shown in Fig. 5(a), CHBr_2_, Br, CH_3_OCHBr_2_, and HBr are present at the onset of the reaction (100 ps). The concentration of Br decays on the nanosecond time scale with a concomitant increase in the concentration of Br_2_, indicating that Br_2_ is formed through a nongeminate recombination of Br, with a bimolecular reaction rate constant of 7.3 ± 1.0 × 10^9^ M^−1^ s^−1^ as shown in Table S5. The concentrations of CH_3_OCHBr_2_ and HBr stay constant until they further increase at ∼40 ns, indicating that these species remain stable over the entire time interval of our measurement and the formation of these species occurs biphasically. We note that the increase in the concentrations of HBr and CH_3_OCHBr_2_ at ∼40 ns is accompanied by the decrease in the concentration of CHBr_2_. We also noted that the amount of consumed Br is larger than the amount of formed Br_2_. These observations suggest that nongeminate association of CHBr_2_ and Br at 40 ns generates *iso-*CHBr_2_-Br, which promptly reacts with methanol to form CH_3_OCHBr_2_ and HBr. The association of CHBr_2_ and Br to form *iso-*CHBr_2_-Br is energetically uphill, yet the solvolysis is strongly downhill, resulting in the overall exothermic reaction. According to our DFT calculations, the reaction barrier for the formation of *iso-*CHBr_2_-Br would be prohibitively high, and we suggest that the overall reaction is solvent-assisted.

Although the concentration profiles determined by TRXL [Fig. 5(a)] and XTA [Fig. 5(b)] are not identical because of the different concentrations of CHBr_3_ solutions used for these two measurements, they share a common set of rate constants. We note that *iso*-CHBr_2_-Br is absent in the kinetics extracted from either TRXL [Fig. 5(a)] or XTA [Fig. 5(b)]. The absence of *iso-*CHBr_2_-Br does not necessarily mean that it is not formed, but it may suggest that *iso-*CHBr_2_-Br has transformed into other species within our time resolution (∼100 ps), probably via the solvolysis reaction with methanol to generate CH_3_OCHBr_2_ and HBr. Indeed, a previous study on the photochemical reaction of CHBr_3_ in water using transient resonance Raman spectroscopy^3^ revealed that *iso-*CHBr_2_-Br reacts rapidly with H_2_O to generate CHBr_2_OH and HBr via hydrolysis and thus was not detected on the picosecond time scale. In this study, CH_3_OCHBr_2_ and HBr rather than *iso-*CHBr_2_-Br, are observed at the earliest delay time as can be seen in Figs. 5(a) and 5(b), which suggests that HBr and CH_3_OCHBr_2_ were generated via Reaction R3, the solvolysis of *iso-*CHBr_2_-Br with methanol, within 100 ps.

**FIG. 6.**
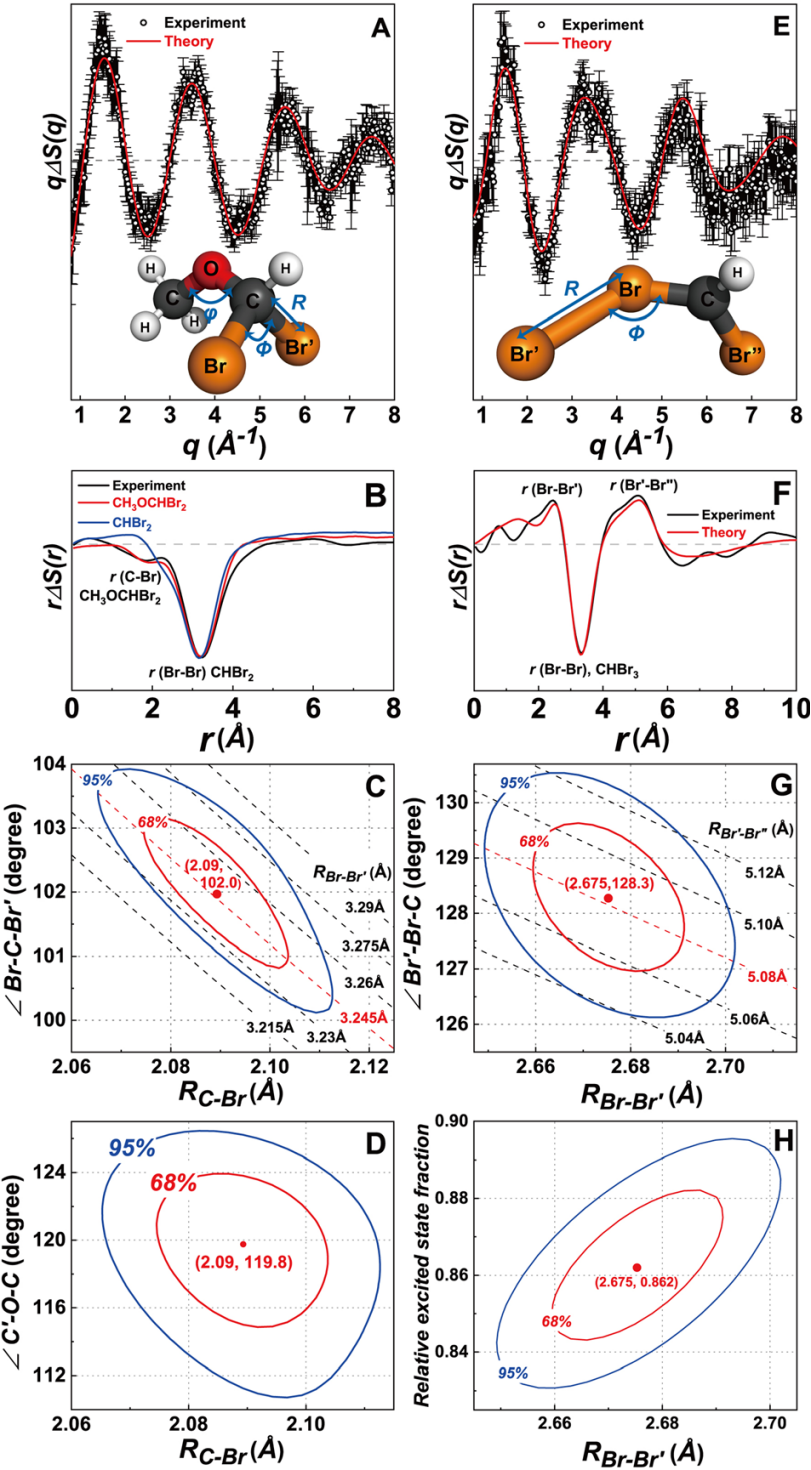
Structural refinement of CH_3_OCHBr_2_ and *iso*-CHBr_2_-Br based on TRXL data. (a) Refined fit (red) of the solute-only TRXL data (dots) for CH_3_OCHBr_2_ at 10 ns. The inset is the molecular structure of CH_3_OCHBr_2_ with the structural fitting parameters: *R*, *ϕ*, and *θ*. (b) Difference radial distribution functions obtained by sine-Fourier transformation of the difference scattering curves shown in (a). The radial distribution function of CHBr_2_ (blue) is shown for comparison. (c) and (d) The figure of merit (*χ^2^*) contour plots of the fitting: The projection of the *χ^2^* surface on (c) (*R, ϕ*) and (d) (*R, θ*) planes. The red and blue ellipses represent statistical confidence areas of 68% and 95%, respectively. The variation of Br–Br′ distance depending on *R* and *ϕ* is shown in panel (c) with dashed lines. The red dots in the contour plots show the optimized values of the parameters. (e) Refined fit (red) of the solute-only TRXL data (dots) for *iso-*CHBr_2_-Br at 100 ps. The inset is the molecular structure of *iso*-CHBr_2_-Br with the structural fitting parameters: *R* and *ϕ*. (f) Difference radial distribution functions obtained by sine-Fourier transformation of the difference scattering curves shown in (e). (g) and (h) The figure of merit (*χ^2^*) contour plots of the fitting: the projection of the *χ^2^* surface on (g) (*R, ϕ*) and (h) (*R, γ*) planes, where *γ* is the relative excited state fraction. The red and blue ellipses represent statistical confidence areas of 68% and 95%, respectively. The variation of the Br′–Br″ distance depending on *R* and *ϕ* is shown in panel (g) with dashed lines.

Our kinetic analysis shows that CH_3_OCHBr_2_ is an important photoproduct that survives even until late delay times in methanol as shown in Figs. 5(a) and 5(b). In Fig. 4, we showed that the formation of CH_3_OCHBr_2_ is required to obtain a good fit to the experimental XTA and TRXL data at both early and late delay times. To determine the structure of CH_3_OCHBr_2_, in Fig. 6(a), we show the solute-only (specifically, CH_3_OCHBr_2_-only) difference scattering curve at 10 ns obtained by subtracting the solvent and cage terms and the contributions of other reaction channels from the experimental difference scattering curve. For comparison, we also show the theoretical difference scattering curve calculated for CH_3_OCHBr_2_, which gives an excellent agreement with the experimental curve. Their corresponding radial intensities are shown in Fig. 6(b). The large negative dip at 3.25 Å corresponds to the depletion of the Br···Br interatomic pair in CHBr_3_. The small negative dip at 1.8 Å and a small peak at 2.2 Å are caused by a superposition of the C-Br distance of 1.9 Å in the depleted CHBr_3_ and the prolonged C–Br distance of 2.09 Å in the generated CH_3_OCHBr_2_, which is a unique signature of CH_3_OCHBr_2_. The C–Br distances in other intermediates are close to the C–Br distance in the parent CHBr_3_ molecule and thus, in contrast to the feature of CH_3_OCHBr_2_, the C-Br features of the other intermediates are canceled out when the reference scattering curve of CHBr_3_ is subtracted, as demonstrated by the theoretical difference scattering curve of CHBr_2_ in Fig. 6(b). The XTA spectra further confirm that CH_3_OCHBr_2_ is the major photoproduct in methanol at late delay times. In Fig. 3, the XTA spectrum at 20 *μ*s is compared with the calculated XTA spectra of CH_3_OCHBr_2_ and CHBr_2_, and it can be seen that CH_3_OCHBr_2_ alone can reproduce most of the features in the experimental XTA spectrum.

### TRXL and XTA measurements in methylcyclohexane

**FIG. 7.**
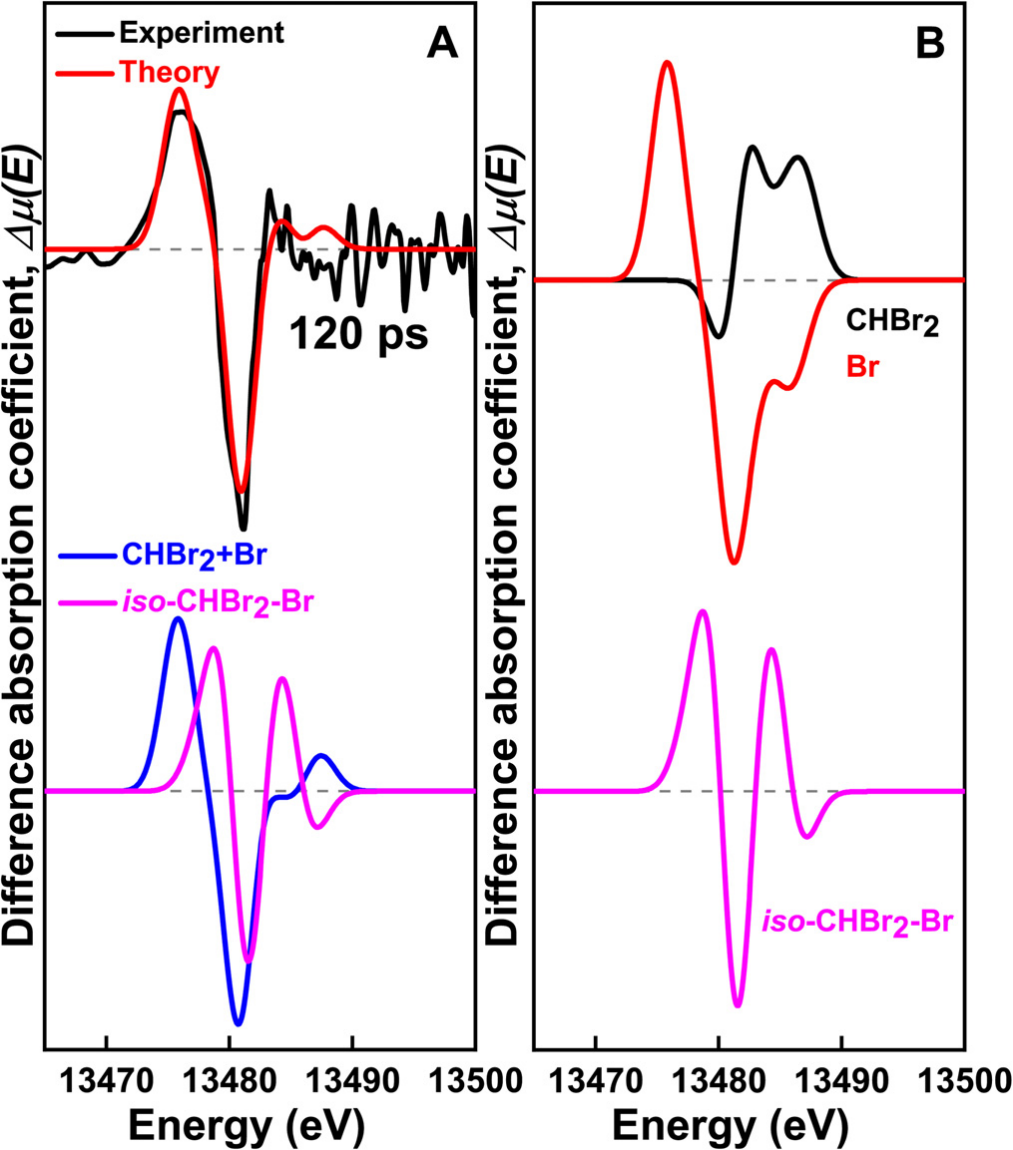
XTA signals measured for CHBr_3_ in methylcyclohexane after 263 nm laser excitation. (a) (Top) XTA spectrum at 120 ps (black) fitted by the calculated spectra (red) considering two reaction channels: (R1) (CHBr_3_ → CHBr_2_ + Br) and (R2) (CHBr_3_ → *iso-*CHBr_2_-Br). (Bottom) Calculated spectra for (R1) (blue) and (R2) (magenta). (b) Calculated XTA signals of the chemical species considered for global fitting.

The presence of O–H bonds in the solvent is essential to drive a solvolysis reaction.^3^ In aprotic solvents, O–H bonds are absent and the solvolysis is not expected to occur, thus presumably leading to the increase in the lifetime of *iso-*CHBr_2_-Br. Indeed, previous studies showed that the lifetime of *iso*-CHBr_2_-Br depends strongly on the solvent properties. In nonpolar solvents such as methylcyclohexane and cyclohexane, *iso*-CHBr_2_-Br survives up to several nanoseconds to microseconds,^4,5^ but it decays rapidly within 13 ps in neat bromoform^4^ and within 1 ns in polar solvents such as acetonitrile.^5^ To determine the structure of *iso*-CHBr_2_-Br in solution, we also studied the photochemistry of CHBr_3_ in methylcyclohexane using TRXL and XTA. Figure 2 shows the TRXL and XTA data of CHBr_3_ in methylcyclohexane at various delay times together with the least squares fit. The same analysis protocol used for the data in methanol was applied to the data in methylcyclohexane. Figure S7 in the supplementary material and Fig. 7 show the analysis results of the TRXL and XTA data, respectively. The analyses of these data as shown in Figs. 7 and S8 indicate that the isomer formation channel is required to obtain a satisfactory fit to both experimental XTA and TRXL data in methylcyclohexane.

Time-dependent concentration changes of various photoproducts were extracted from the simultaneous GF analyses of the TRXL and XTA data as shown in Figs. 5(c) and 5(d), respectively. According to the kinetics determined from the TRXL data, at the onset of the reaction, the CHBr_2_ radical, Br, and *iso*-CHBr_2_-Br coexist, which is in contrast to the reaction in methanol where *iso*-CHBr_2_-Br is absent. Then, *iso*-CHBr_2_-Br returns to CHBr_3_ in 73 ns with a unimolecular rate constant of 7.7 ± 1.6 × 10^6^ s^−1^. The Br atoms recombine to generate Br_2_ with a bimolecular rate constant of 5.04 ± 0.7 × 10^8^ M^−1^s^−1^, which is smaller than that in methanol by an order of magnitude. CHBr_2_ stays stable on the time scale of microseconds.

As was the case for the result in methanol, the XTA data also provide direct information on the reaction dynamics, as shown in Fig. 2(c) and S9 in the supplementary material. The positive peak at 13 475 eV at early delay times corresponds to the 1s → 4p transition of Br and arises from the formation of Br, which disappears at late delay times when Br atoms react to form other species. In contrast to the reaction in methanol, the negative peak in the difference XTA spectra in methylcyclohexane stays at the same energy throughout the time interval while its intensity decreases by about a factor of two at late delay times, suggesting that ∼50% of the photoproducts return to CHBr_3_ (Fig. S9). Indeed, in methylcyclohexane, *iso*-CHBr_2_-Br accounts for about half of the photoproducts and relaxes back to CHBr_3_ in tens of nanoseconds [Fig. 5(d)], while the recovery of CHBr_3_ was not observed in methanol in the time range of our measurements.

To better visualize the formation of *iso-*CHBr_2_-Br in methylcyclohexane at the onset of the reaction, the contributions of the solvent and cage terms as well as the contribution of reaction (R1) were subtracted from the difference X-ray scattering data at 100 ps. The resulting solute-only (specifically, *iso*-CHBr_2_-Br-only) difference scattering curve and its difference radial intensity agree well with the theoretical scattering curve and its radial intensity calculated for the isomerization reaction as shown in Figs. 6(e) and 6(f), respectively, confirming the formation of *iso-*CHBr_2_-Br. In the difference radial intensities shown in Fig. 6(f), the large negative dip at 3.25 Å corresponds to the depletion of the Br···Br interatomic distance in CHBr_3_ while the positive peaks at 2.5 Å and 5.1 Å arise from the Br–Br′ and Br′···Br″ interatomic pairs, respectively, which are characteristic of *iso*-CHBr_2_-Br.

### Structural refinement of CH_3_OCHBr_2_ in methanol and *iso-*CHBr_2_-Br in methylcyclohexane

We refined the geometric structures of CH_3_OCHBr_2_ in methanol and *iso-*CHBr_2_-Br in methylcyclohexane using the TRXL data, as shown in Fig. 6. The structural parameters were optimized together with the excited state fraction (*γ*) to compensate for the reduction of the amplitude of the difference scattering curve as the structural parameters are varied. The confidence intervals and correlations of all parameters were obtained from the *χ^2^* contour plots as explained in Sec. 4 in the supplementary material.

For CH_3_OCHBr_2_, we optimized the C–Br bond length (*R*), Br–C–Br′ angle (*ϕ*), and C′–O–C angle (*Θ*) [see the inset in Fig. 6(a)]. To do so, we used the solute-only difference scattering curve at 10 ns, as shown in Fig. 6(a). Variation of either *R* or *ϕ* changes the Br-Br′ distance, indicating a strong negative correlation between *R* and *ϕ* [Fig. 6(c)]. On the other hand, *Θ* has a much weaker correlation with both *R* and *ϕ* [see Figs. 6(d) and S10] because the methyl group consists of relatively light atoms compared with Br and therefore the change of its absolute position influences the simulated difference scattering signal only slightly. The optimal values for *R*, *ϕ*, and *Θ* within the 68% confidence are *R *=* *2.09 ± 0.02 Å, *ϕ* = 102 ± 1°, and *Θ *=* *119.8 ± 4° [see Fig. 6(c)]. The former two parameters define the optimal Br-Br′ distance to be 3.245 ± 0.02 Å. Further details of the structural refinement are described in Sec. 4 in the supplementary material. The optimized bond lengths and angles are in good agreement with our DFT calculations, as shown in Table I.

**TABLE I.** Refined bond lengths (angstrom) and angles (degree) of CH_3_OCHBr_2_ in methanol and *iso*-CHBr_2_-Br in methylcyclohexane determined from TRXL data, and their comparison with the DFT calculations.

Species	Structural parameters	Experiment	DFT
CH_3_OCHBr_2_^a^	C-Br:	2.09 ± 0.02	2.01
	∠C′OC:	119.8 ± 4	120.02
	Br···Br′:	3.24 ± 0.02^b^	3.26
	∠BrCBr′:	102.0 ± 1	108.29
*iso*-CHBr_2_-Br^a^	Br-Br′:	2.68 ± 0.02	2.77
	Br′···Br″:	5.08 ± 0.02	5.14
	∠Br′BrC:	128 ± 1	127.5

^a^ Labels and symbols are referenced to the insertion structures in Figs. 6(a) and 6(e), respectively.

^b^ The error of Br···Br′ was estimated based on the uncertainties of the C-Br distance and BrCBr′ angle.

For *iso-*CHBr_2_-Br, the Br–Br′ bond length (*R*) and Br′–Br–C angle (*ϕ*) were refined [see the inset in Fig. 6(e)]. To do so, we used the solute-only difference scattering curve at 100 ps, as shown in Fig. 6(e). As can be seen in Figs. 6(g) and 6(h), strong correlations are observed for both (*R*, *ϕ*) and (*R,* γ) pairs of parameters because the changes of *R* or *ϕ* directly affect the Br′–Br″ distance. The refined values of *R* and *ϕ* with 68% confidence are *R *=* *2.68 ± 0.02 Å and *ϕ* = 128 ± 1°, which results in the Br′–Br′ distance of 5.08 ± 0.02 Å (see Table I).

### Reaction energetics and thermodynamics of the bulk solvent

As the photogenerated intermediate species recombine back to the ground state, excess energy is released as heat to the surrounding solvent, resulting in changes of temperature, pressure, and density of the solvent, and ultimately the variation of intermolecular radial distributions in the solvent.^19^ Although for a single solvent molecule, this variation might be negligible, given the large relative amount of solvent molecules over solutes (with a concentration of 40 mM in methanol as an example, the ratio between the number of solute and solvent molecules is 1:618), the integrated solvent signal can be comparable to or even larger than the solute-only signal. In the difference X-ray scattering signals, the transient solvent response typically dominates at late delay times after the intermediate molecules relax back to the ground state, and it has a large amplitude at low to medium *q* values or in a high *r*-region, as shown in Figs. S1 and S7. In the GF analysis, the time dependence of the thermodynamic quantities such as the changes in density Δρ(t) and temperature ΔT(t) of the bulk solvent can be monitored as the heat is released from the solutes and propagates through the solution. Figure S11 shows the temporal variations of Δρ(t) and ΔT(t) in methanol and methylcyclohexane after laser excitation.

### Stable photoproducts

We identified the stable photoproducts by analyzing the photolyzed solution of CHBr_3_ in methanol (see Secs. 5 and 6 in the supplementary material for the details). First, we noticed that the transparent CHBr_3_/methanol solution changed to orange color during the experiment (Fig. S12), which can be ascribed to the formation of stable Br_2_ as confirmed by the electronic absorption spectra measured at UV to visible wavelengths (Fig. S13). Also, from the analysis of the photolyzed solution with GCMS and NMR spectroscopy, we found that CH_2_Br_2_, BrCH_2_OH, C_2_H_2_Br_4_, *cis*-C_2_H_2_Br_2_, and *trans*-C_2_H_2_Br_2_ are formed with comparable yields (see Table S3 and Figs. S15–S27 in the supplementary material) from various reactions following the photoexcitation of CHBr_3_. For example, CH_2_Br_2_ was formed through the following abstraction reaction:

$${\text{CHBr}}_{2}+{\;\text{CH}}_{3}\text{OH}\;\to {\;\text{CH}}_{2}{\text{Br}}_{2}+{\;\text{CH}}_{2}\text{OH},$$ (R4)

as was confirmed by the formation of CHDBr_2_ from the photolysis of CHBr_3_ in CD_3_OD. It should be noted that CH_2_Br_2_, which is observed from the photolysis of CHBr_3_ in methanol, is not observed in methylcyclohexane, confirming that CH_2_Br_2_ is formed via the abstraction reaction [reaction (R4)]. The identification of these various photoproducts is discussed in detail in Secs. 5 and 6 in the supplementary material. Including these species in the GF analysis does not improve the fitting quality significantly, and the concentration of these species is below the threshold of the signal-to-noise ratio of our experimental data. Our data analysis illustrates that these stable photoproducts are probably formed on longer time scales, and the secondary photochemistry of CHBr_3_ involves numerous inter-related reactions.

## CONCLUSION

**FIG. 8.**
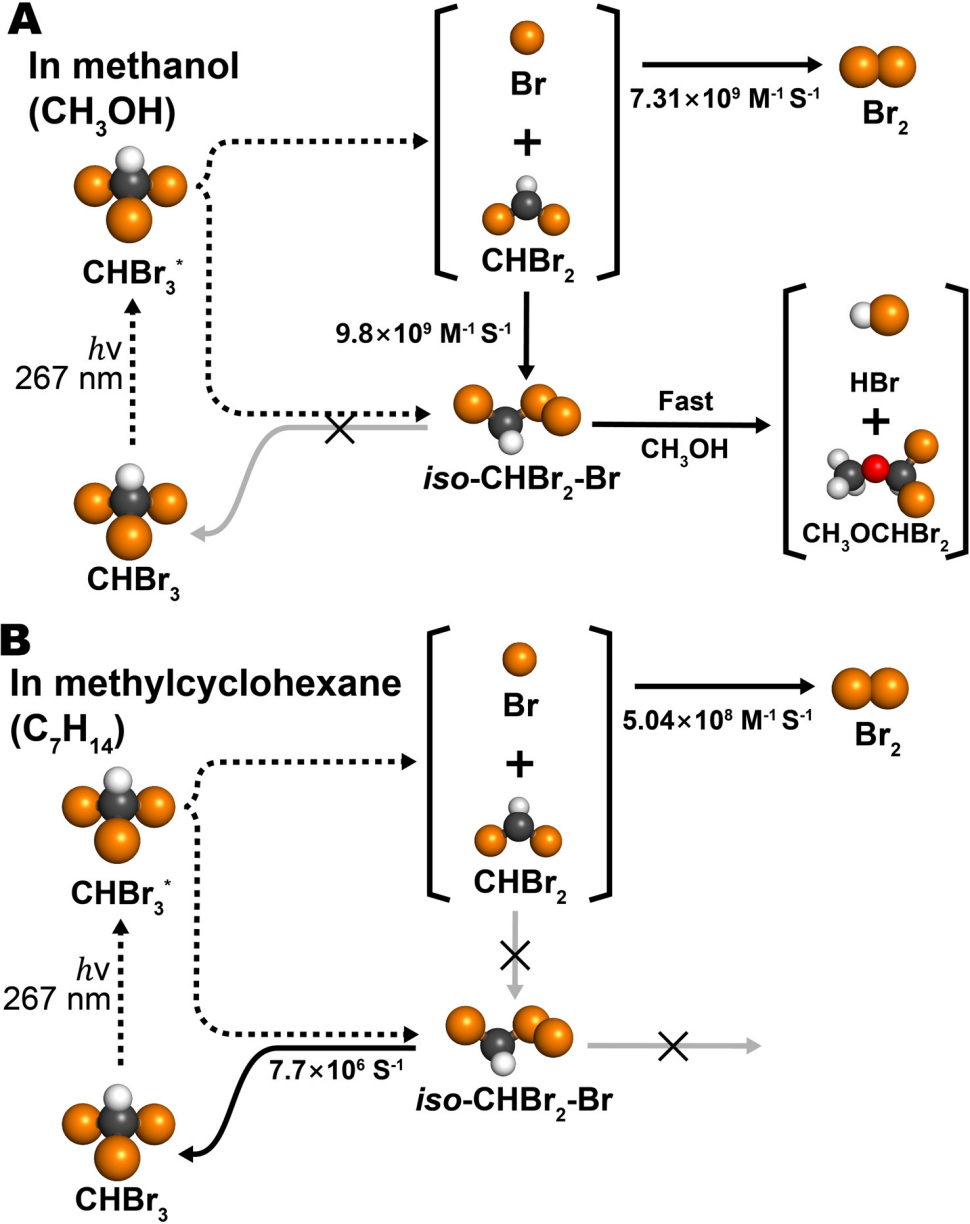
Reaction mechanisms for the photochemistry of CHBr_3_ in methanol and methylcyclohexane. Bromine, carbon, hydrogen, and oxygen atoms are shown in orange, black, white, and red, respectively.

Complex photochemical reaction pathways of CHBr_3_ in methanol and methylcyclohexane were elucidated using picosecond TRXL and XTA. The homolytic C–Br bond cleavage leading to the formation of the CHBr_2_ radical, Br, and Br_2_ is a common reaction channel observed in both solvents. In methanol, the rapid solvolysis of *iso*-CHBr_2_-Br [reaction (R3)] yields CH_3_OCHBr_2_ and HBr at 100 ps, which survive for tens of microseconds. In contrast, in methylcyclohexane, *iso-*CHBr_2_-Br does not undergo solvolysis and instead isomerizes back to CHBr_3_ with a time constant of ∼70 ns, which is specific to the concentration and excitation energy used in our experiment. Our study illustrates the amazing complexity of the photochemical reaction pathways of CHBr_3_, which vary with the solvent properties, as schematically shown in Fig. 8. Besides this complexity, the combination of two time-resolved X-ray techniques proved to be capable of providing a complete description for all major photochemical reaction pathways for this environmentally important molecule.

## SUPPLEMENTARY MATERIAL

See the supplementary material for the details of experimental methods, data analysis process, and characterization of stable photoproducts.
